# Influence of Pathogenic and Metabolic Genes on the Pharmacogenetics of Mood Disorders in Alzheimer’s Disease

**DOI:** 10.3390/ph14040366

**Published:** 2021-04-15

**Authors:** Ramón Cacabelos, Juan C. Carril, Lola Corzo, Lucía Fernández-Novoa, Rocío Pego, Natalia Cacabelos, Pablo Cacabelos, Margarita Alcaraz, Iván Tellado, Vinogran Naidoo

**Affiliations:** International Center of Neuroscience and Genomic Medicine, EuroEspes Biomedical Research Center, 15165-Bergondo, Corunna, Spain; genomica@euroespes.com (J.C.C.); analisis@euroespes.com (L.C.); genetica@ebiotec.com (L.F.-N.); neuropsicologia@euroespes.com (R.P.); serviciodocumentacion@euroespes.com (N.C.); asistentedireccion@euroespes.com (P.C.); enfermeria@euroespes.com (M.A.); diagnosticodigital@euroespes.com (I.T.); neurociencias@euroespes.com (V.N.)

**Keywords:** Alzheimer’s disease, anxiety, APOE, CYP2D6, CYP2C9, CYP2C19, depression, genomics, mood disorders, pharmacogenomics

## Abstract

Background: Mood disorders represent a risk factor for dementia and are present in over 60% of cases with Alzheimer’s disease (AD). More than 80% variability in drug pharmacokinetics and pharmacodynamics is associated with pharmacogenetics. Methods: Anxiety and depression symptoms were assessed in 1006 patients with dementia (591 females, 415 males) and the influence of pathogenic (APOE) and metabolic (CYP2D6, CYP2C19, and CYP2C9) gene variants on the therapeutic outcome were analyzed after treatment with a multifactorial regime in a natural setting. Results and Conclusions: (i) Biochemical, hematological, and metabolic differences may contribute to changes in drug efficacy and safety; (ii) anxiety and depression are more frequent and severe in females than males; (iii) both females and males respond similarly to treatment, showing significant improvements in anxiety and depression; (iv) APOE-3 carriers are the best responders and APOE-4 carriers tend to be the worst responders to conventional treatments; and (v) among CYP2D6, CYP2C19, and CYP2C9 genophenotypes, normal metabolizers (NMs) and intermediate metabolizers (IMs) are significantly better responders than poor metabolizers (PMs) and ultra-rapid metabolizers (UMs) to therapeutic interventions that modify anxiety and depression phenotypes in dementia. APOE-4 carriers and CYP-related PMs and UMs deserve special attention for their vulnerability and poor response to current treatments.

## 1. Introduction

Over 50 million people suffer dementia and it is expected that 75 million people will be affected in 2030 and 145 million in 2050, at an increasing rate of 7.7 million new cases per year. The global economic cost for dementia is over US$604 billion, equivalent to 1% of the global gross domestic product. Alzheimer’s disease (AD) is the most frequent form of dementia (>50%), followed by vascular (VD) and mixed dementia (MXD) (30–40%) and other phenotypic presentations of neurocognitive disorders (NCDs) [1]. In patients over 70–75 years of age, MXD is the most prevalent form of dementia (>70%) [2].

Genomic defects, epigenetic aberrations, cerebrovascular damage, and environmental inducers are the major risk factors that precipitate pathogenic cascades leading to the clinical phenotype of dementia. Dementia is characterized by progressive cognitive deterioration, behavioral changes, functional decline, and classical neuropathological hallmarks represented by extracellular Aβ deposition in senile plaques, intracellular neurofibrillary tangles, aberrant dendritic morphology, and neuronal loss in critical regions of the central nervous system (CNS) [3,4,5,6,7].

Behavioral changes, such as psychotic, depressive and anxiety symptoms, sleep disorders and inappropriate behaviors are present in 10–90% of patients with dementia, and these behavioral disorders (BDs) tend to increase in parallel with the cognitive deterioration [8,9,10,11,12,13]. BDs also contribute to accelerated cognitive decline, impair daily functioning, reduce the quality of life, and increase the risk of institutionalization [14,15], magnifying the costs of dementia care [16]. Furthermore, some neuropsychiatric disorders may increase the risk for late-onset dementia, and dementia may increase the risk for delayed-onset BDs [17]. Over 50% of AD patients have comorbidities, and the presence of gene variants, together with metabolic disorders, cerebrovascular risk, premorbid personality, and inappropriate management may contribute to BDs in AD [2]. Although there is no prototypical pattern of BDs in different types of dementia, anxiety, depression, apathy, dysphoria, agitation, aggression, delusions, and hallucinations are frequent distressing symptoms in dementia that require pharmacological intervention [2,13].

Conventional treatments for AD include acetylcholinesterase inhibitors (donepezil, rivastigmine, galantamine) and memantine, as well as other cognitive enhancers and concomitant treatments [18]. No FDA-approved drugs for AD have been reported for the past two decades [19]. Most BDs in dementia are susceptible to treatment with psychotropic medication. Inappropriate treatments, drug–drug interactions (DDIs), and adverse drug reactions (ADRs) are frequent and deleterious in this fragile population [20,21,22,23]. Patients with dementia receive 6–12 different drugs per day; and current ADRs in the elderly are associated with benzodiazepines, neuroleptics, antidepressants, and specific treatments for concomitant disorders (e.g., cardiovascular, respiratory, hypertension, diabetes, dyslipidemia, pain, etc.) [18,23,24,25,26].

About 80% variability in drug efficacy and safety is attributed to pharmacogenetics [24,25,26,27,28,29,30,31,32,33,34,35,36,37]. Rare variants contribute to 50% of functional variability in over 140 pharmagenes with clinical relevance. Over 400 genes and their encoded proteins/enzymes influence drug efficacy and safety; and about 240 pharmagenes with uneven influence are potentially associated with ADRs [28,29]. The pharmacological outcome is highly influenced by components of the pharmacogenetic machinery, the chemical properties of each drug, and other diverse factors (e.g., compliance, nutrition, metabolic conditions, and concomitant drugs) [30,31,32,33]. The pharmacogenetic machinery is integrated by pathogenic, mechanistic, metabolic, transporter, and pleiotropic genes under the promiscuous regulation of epigenetic factors such as DNA methylation, chromatin/histone changes, and miRNAs [32,33]. In AD, most pharmacogenetic studies used apolipoprotein E (APOE) and cytochrome P450 (CYP) variants, as a reference, since the presence of the APOE-4 allele is a major pathogenic risk factor for dementia and most acetylcholinesterase inhibitors are metabolized via CYP enzymes, with the exception of rivastigmine [2,4,6,18,34,35]. In general, APOE-3 carriers are the best responders and APOE-4 carriers are the worst responders to treatments either in monotherapy or in combination regimes [2,4,6,18,34,35]. Among the CYP variants, CYP2D6 normal metabolizers (NM) tend to be the best responders to treatment, CYP2D6 intermediate metabolizers (IM) show an intermediate response, and CYP2D6 poor metabolizers (PM) and ultra-rapid metabolizers (UM) tend to behave as the worst responders to conventional anti-AD treatments [2,4,6,18,34,35,36,37].

The prevalence of depression in AD ranges from 5% to >40% in various studies, and depression is the second most common psychiatric symptom in AD, after apathy. Severe cases of AD exhibit a higher prevalence of depression [38,39], and the presence of depressive symptoms is associated with a faster rate of memory decline [40]. Late-life depression may represent a prodrome to dementia [41], with sex-specific differences [42,43]; and depressed patients with mild cognitive impairment (MCI) exhibit worse cognitive performance and greater loss of gray-matter volume [44]. Furthermore, cardiovascular risk factors have been proposed as an additional inducer of depression in patients with MCI [45].

Anxiety-like symptoms occur in 30–40% of cases with dementia. Anxiety is also a risk factor for dementia [46,47] and anxiety-like behaviors are persistent in patients with dementia. Early-onset AD patients exhibit greater prevalence of all BDs, especially anxiety, irritability, and sleep disorders [48].

In this retrospective study, we investigate the presence of anxiety and depression in a well-characterized sample of AD patients and analyzed the influence of APOE variants and CYP2D6, CYP2C19, and CYP2C9 geno-phenotypes on the frequency and severity of anxiety and depression as well as the effect that different APOE single nucleotide polymorphisms (SNPs) and CYP phenotypes exert on the evolution of mood disorders after treatment with multifactorial therapy.

## 2. Results

### 2.1. Sex-Related Biochemical, Hematological, Metabolic and Clinical Phenotypes

Age was almost identical in females and males ranging from 50–96 years. However, globally, biochemical, hematological, metabolic, psychometric, and anthropometric parameters were significantly different between females and males (Table 1). The electrocardiogram (EKG) was abnormal in 47.82% of cases (43.36% in females and 54.35% in males, *p* < 0.001). Systolic and diastolic pressure values were higher in males than females (*p* < 0.001); however, heart rate was faster in females than males (*p* < 0.001). Total cholesterol, HDL-cholesterol, and LDL-cholesterol levels were also higher in females than males (*p* < 0.001), and triglycerides were higher in males than females (*p* < 0.005). Glucose levels were much higher in males than females (*p* < 0.001). Other important differences were found in kidney function, liver function, creatine phosphokinase (CK), alkaline phosphatase (ALP), and electrolytes (K^+^). Red blood cell, hematocrit, and hemoglobin values were higher in males than females whereas platelets were more elevated in females than males. White blood cell number was also higher in males than females, especially leukocytes, monocytes, basophils, and eosinophils, while lymphocytes were slightly more abundant in females. Iron and ferritin values were higher in males; in contrast, folic acid and vitamin B12 values were higher in females (Table 1).

### 2.2. Cognition

The Basal Mini-Mental State Examination (MMSE) score was 23.16 ± 5.95 (females: 22.42 ± 5.88; males: 24.22 ± 5.90, *p* < 0.001) and the AD Assessment Scale (ADAS)-Total score was 22.78 ± 15.22 (females: 23.76 ± 15.07; males: 21.36 ± 15.35, *p* < 0.002). Cognitive function improved for the first 6 months with this therapeutic regime and declined thereafter, as reported elsewhere [4,6,34,36,37,49].

### 2.3. Anxiety

**Figure 1:**
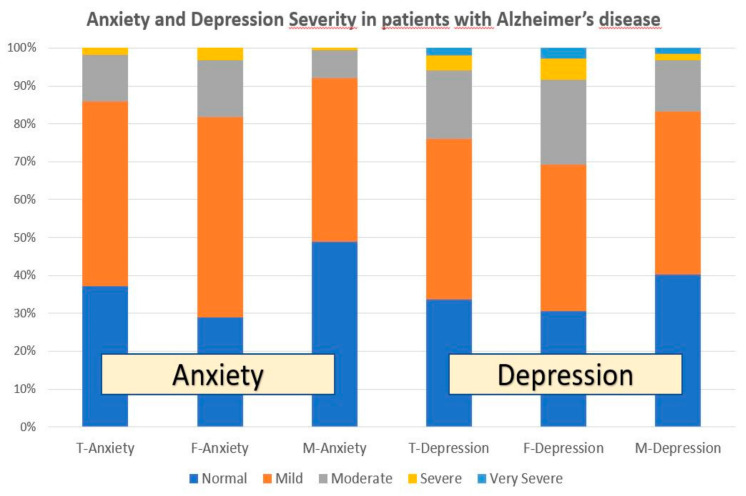
Anxiety and depression in patients with AD. T: Total sample; F: Females; M: Males.

**Figure 2:**
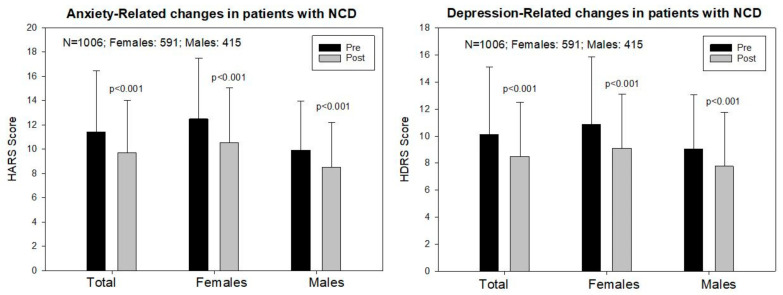
Sex-related anxiety and depression responses to a multifactorial treatment in patients with Alzheimer’s disease.

**Figure 3:**
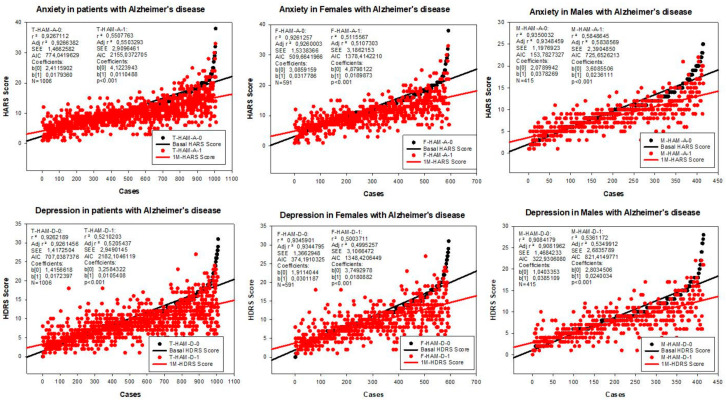
Correlation analysis of the therapeutic response of females and males with AD to a combination treatment. F: Females; M: Males; HAM-A: Hamilton Anxiety Rating Scale Score; HAM-D: Hamilton Depression Rating Scale Score; A0: Basal HAM-A; A1: HAM-A after 1-month treatment; D0: Basal HAM-D; D1: HAM-D after 1-month treatment.

Anxiety was present in nearly 60% of patients (anxiety-free cases: 37.18%) and was significantly more frequent in females than males (*p* < 0.001). In the global sample, 48.88% of cases showed mild-anxiety (females: 52.79%; males: 43.13%; *p* < 0.001); moderate-anxiety appeared in 12.33% of the cases (females: 15.05%; males: 7.47%; *p* < 0.001); and severe-anxiety was only observed in 1.69% of the cases (females: 3.21%; males: 0.48%; *p* < 0.001) (Figure 1, Figure 2 and Figure 3).

The baseline Hamilton Anxiety Rating Scale (HARS) score was 11.44 ± 5.41 in the total sample, 12.49 ± 5.63 in females, and 9.94 ± 4.69 in males (*p* < 0.001); and a significant improvement was observed following one month of multifactorial treatment in both sexes (T: 9.79 ± 4.33, *p* < 0.001; F: 10.53 ± 4.55, *p* < 0.001; M: 8.52 ± 3.70, *p* < 0.001) (Figure 2 and Figure 3).

### 2.4. Depression

Depressive symptoms were present in almost 70% of the cases (depression-free cases: 33.70%), and depression was also more frequent in females than males (*p* < 0.05). In the global sample, 42.34% of the patients showed mild-depression (females: 38.62%; males: 42.89%; *p* = 0.28); 18.19% moderate-depression (females: 22.46%; males: 13.73%; *p* < 0.001); 3.78% severe-depression (females: 5.51%; males: 1.69%; *p* < 0.001); and 1.99% very severe-depression (females: 2.85%; males: 1.45%; *p* = 0.07) (Figure 1 and Figure 3).

The baseline Hamilton Depression Rating Scale (HDRS) score was 10.11 ± 5.21 in the total sample, 10.85 ± 5.33 in females, and 9.05 ± 4.85 in males (*p* < 0.001). One month later, a significant improvement was observed in the total group (8.57 ± 4.25, *p* < 0.001), and in both females (9.12 ± 4.39) and males (7.79 ± 3.93, *p* < 0.001) (Figure 2 and Figure 3).

### 2.5. Pharmacogenomics

**Figure 4:**
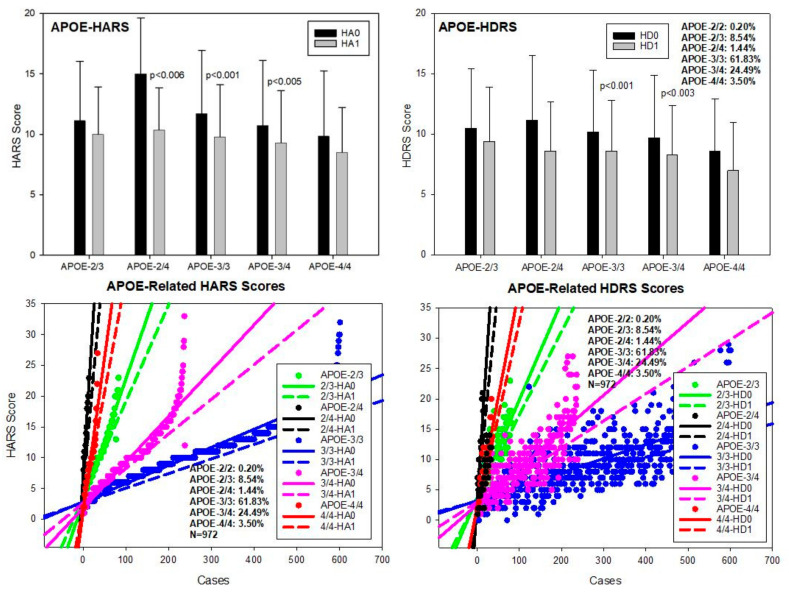
APOE-related anxiety and depression in patients with AD. HA0: Basal Hamilton Anxiety Rating Scale Score; HA1: HARS score after 1-month treatment. HD0: Basal Hamilton Depression Rating Scale Score; HD1: HDRS score after 1-month treatment.

**APOE-E**. The distribution and frequency of APOE genotypes were the following: APOE-2/2 0.20%, APOE-2/3 8.54%, APOE-2/4 1.44%, APOE-3/3 61.83%, APOE-3/4 24.49%, and APOE-4/4 3.50% (Table 2 and Table 3). Baseline values of anxiety (HARS-0) were significantly different between APOE-2/3 and 2/4 (*p* < 0.007); APOE-2/4 vs. 3/3 (*p* < 0.01), vs. 3/4 (*p* < 0.002), and vs. 4/4 (*p* < 0.001); and APOE-3/3 vs. 3/4 (*p* < 0.005) and vs. 4/4 (*p* < 0.02) (Table 2; Figure 4). Improvement in anxiety symptoms was observed in most APOE variants; however, the best responders to multifactorial intervention were APOE-3/3 (*p* < 0.001), APOE-3/4 (*p* < 0.001), and APOE-2/4 carriers (*p* < 0.006) (Table 2; Figure 4). Baseline HDRS values for depression were significantly different between APOE-2/3 and 3/4 (*p* < 0.05) and 2/3 vs. 4/4 (*p* < 0.05); and also between APOE-2/4 and 4/4 (*p* < 0.05) and between APOE-3/3 and 4/4 (*p* < 0.05) (Table 3; Figure 4). The best responders to treatment were APOE-3/3 (*p* < 0.001) and APOE-3/4 carriers (*p* < 0.003) (Table 3; Figure 4).

**Figure 5:**
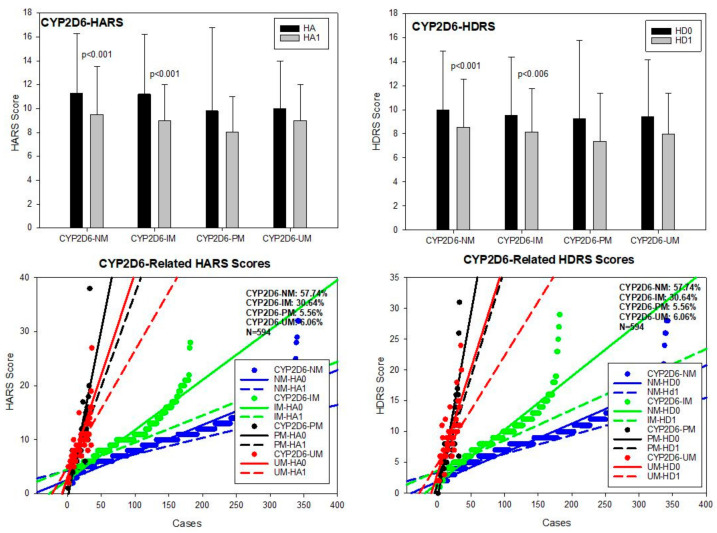
CYP2D6-related anxiety and depression in patients with AD. HA0: Basal Hamilton Anxiety Rating Scale Score; HA1: HARS score after 1-month treatment. HD0: Basal Hamilton Depression Rating Scale Score; HD1: HDRS score after 1-month treatment. NM: CYP2D6 Normal Metabolizers; IM: Intermediate Metabolizers; PM: Poor Metabolizers; UM: Ultra-Rapid Metabolizers.

**CYP2D6**. The most frequent CYP2D6 variants in our population were CYP2D6*1A (PV00126)(wild type), *3A (PV00221)(2550delA)(rs35742686), *4 (PV00235)(1847G > A)(rs3892097), *5 (PV00259)(gene deletion), *6A (PV00198)(1708delT)(rs5030655), *41 (PV00239)(2989G > A)(rs28371725), and *1 × N (gene duplication, multiplication). Major genotypes included CYP2D6*1A/*1A, *1A/*3A, *1A/*4A, *1A/*5, *1A/*6A, *1A/*41, *3A/*3A, *3A/*4A, *3A/*6A, *4A/*4A, *4A/*5, *4A/*6A, *4A/*41, *5/*5, *5/*6A, *41/*41, *1A/*1 × N, *3A/*1 × N, *4A/*1 × N, *5/*1 × N, and *41/*1 × N, conferring the condition of normal metabolizer (NM) (57.74%), intermediate metabolizer (IM) (30.64%), poor metabolizer (PM) (5.56%), and ultra-rapid metabolizer (UM) (6.06%) phenotypes. Baseline HARS scores were similar among CYP2D6 geno-phenotypes, except between CYP2D6-NM and PM (*p* < 0.03) and between CYP2D6-IM and PM (*p* < 0.05). All CYP2D6 geno-phenotypes responded similarly to treatment; however, the best responders were CYP2D6-NM (*p* < 0.001) and IM (*p* < 0.001) (Table 2; Figure 5). Baseline HDRS scores did not show any difference among CYP2D6 geno-phenotypes. Although all variants improved with treatment, only CYP2D6-NM and IM showed significant differences as compared with basal HDRS score values, following a similar yet more modest pattern to that observed for anxiety (Table 3; Figure 5).

**Figure 6:**
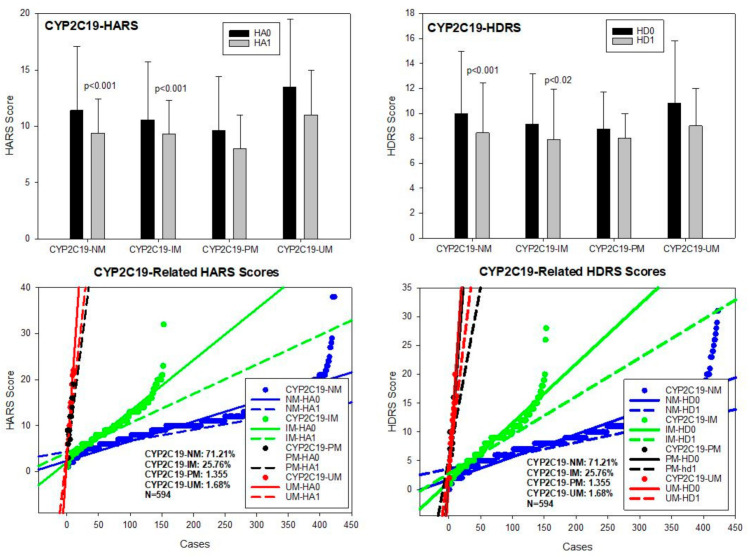
CYP2C19-related anxiety and depression in patients with AD. HA0: Basal Hamilton Anxiety Rating Scale Score; HA1: HARS score after 1-month treatment. HD0: Basal Hamilton Depression Rating Scale Score; HD1: HDRS score after 1-month treatment. NM: CYP2C19 Normal Metabolizers; IM: Intermediate Metabolizers; PM: Poor Metabolizers; UM: Ultra-Rapid Metabolizers.

**CYP2C19**. The CYP2C19 gene exhibits 541 allelic variants among which the most important alleles were CYP2C19*1A (wild type) (PV00081), 2C19*2A (19154G > A) (PV00111) (rs4244285), and 2C19*17 (−806C > T) (PV00097) (rs12248560). In our sample, the most prevalent geno-phenotypes were CYP2C19*1A/*1A-EM and *2A/*17*-EM (71.21%), *1A/*2A-IM (25.76%), *2A/*2A-PM (1.35%), and *1A/*17-UM and *17/*17-UM (1.68%) (Table 2; Figure 6). No differences have been found among baseline HARS scores for anxiety, and only CYP2C19-NM (*p* < 0.001) and IM (*p* < 0.01) significantly responded to treatment. CYP2C19-PM and UM showed a mild, non-significant improvement in anxiety symptoms (Table 2; Figure 6). Basal HDRS scores showed differences between CYP2C19-NM and IM (*p* < 0.05) and between CYP2C19-NM and PM (*p* < 0.05). Improvement in depression HDRS scores were observed in CYP2C19-NM (*p* < 0.001) and IM (*p* < 0.02), with no response in CYP2C19-PM and minimum improvement in CYP2C19-UM (Table 3; Figure 6).

**Figure 7:**
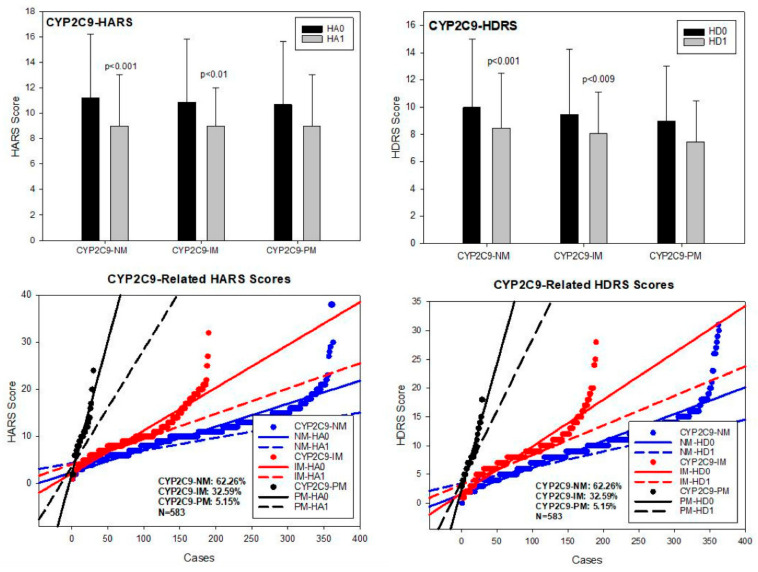
CYP2C9-related anxiety and depression in patients with AD. HA0: Basal Hamilton Anxiety Rating Scale Score; HA1: HARS score after 1-month of treatment. HD0: Basal Hamilton Depression Rating Scale Score; HD1: HDRS score after 1-month of treatment. NM: CYP2C9 Normal Metabolizers; IM: Intermediate Metabolizers; PM: Poor Metabolizers.

**CYP2C9**. Among the 480 allelic variants identified in the CYP2C9 gene, the most relevant alleles were 2C9*1A (wild type) (PV00030), 2C9*2A (3608C > T) (PV00044) (rs1799853), 2C9*3A (47639A > C) (PV00058)(rs1057910), and 2C9*5A (476aaC > G)(PV00025) (rs28371686). Major CYP2C9 geno-phenotypes in our sample were CYP2C9*1A/*1A-EM (62.26%), *1A/*2A-IM and *1A/*3A-IM (32.59%), and *2A/*2A-PM, *2A/*3A-PM and *3A/*3A-PM (5.15%) (Table 2; Figure 7). No differences in either basal HARS and HDRS scores were found. Both CYP2C9-NM and IM responded to treatment with a significant improvement in anxiety and depression symptoms (*p* < 0.001), and CYP2C9-PM experienced a mild, non-significant improvement in both depression and anxiety HDRS and HARS scores, respectively (Table 3; Figure 7).

## 3. Discussion

### 3.1. Parametric Differences

Mood disorders in dementia are the result of a number of factors, either endogenous or exogenous. Our study clearly shows substantial sex-related differences in the frequency and intensity of anxiety and depression in AD patients (Table 2 and Table 3; Figure 1 and Figure 3), as well as striking differences in anthropometric, biochemical, hematological, metabolic, and psychometric parameters between both sexes (Table 1, Table 2 and Table 3) that directly and/or indirectly may affect cognition and emotional status. Although mood disorders appear to be an intrinsic component of the dementia behavioral phenotype, in our sample of well-characterized patients, with a clinical follow-up for two decades, the improvement observed in both anxiety and depression cannot be exclusively attributed to psychotropic treatment, but also to the multifactorial intervention, covering concomitant problems, which were present in over 60% of the patients. Mood disorders in our sample appear to be unrelated to cardiovascular factors; and, apparently, both anxiety and depression present with greater frequency, with higher intensity, in females with lower red blood cell parameters and poorer metabolic conditions. Mood disorders are also unrelated to hyperglycemia in dementia (Table 1).

### 3.2. Pharmacogenetic Determinants

Major determinants of the pharmacological outcome in dementia include age, gender, race, nutritional status, drug properties (chemistry, pharmaceutical category, biopharmaceutical properties, drug source: Synthetic, natural), route of administration, dose, pharmacokinetics, pharmacodynamics, drug target(s), disease stage, concomitant treatments, compliance rate, pharmacogenomics, and pharmacoepigenomics [24,25].

Our study corroborates the assertion that APOE is a major, but not exclusive, pathogenic determinant of the pharmacogenetic outcome in AD and that APOE-4 carriers are at biological disadvantage with respect to patients harboring the APOE-3 allele, as previously documented [2,4,6,18,34,35,36,37,49,50,51]. Most studies, where AD patients were treated with multifactorial combinations, revealed that APOE-3/3 carriers are the best responders and APOE-4/4 carriers are the worst responders. Concerning CYP-related pharmacogenomic outcomes, CYP2D6-EMs are the best responders, CYP2D6-PMs are the worst responders, and CYP2D6-IMs and UMs show an intermediate response. Many other pathogenic genes [50] and genes encoding components of the epigenetic machinery [51] also influence the pharmacogenetic outcome. Genome-wide association studies (GWAS) identified 31 genes located in 19 risk loci for major depressive disorder (MDD). Common and rare variants of L3MBTL2 are associated with AD. Transcript (mRNA) expression levels of SORCS3 and OAT are differentially expressed in AD brain tissues, and 13 MDD risk genes may interact with core AD genes such as HACE1, NEGR1, and SLC6A15 [52].

### 3.3. Metabolic Genes

Anxiolytics and antidepressants act as substrates, inhibitors, or inducers of enzymes encoded by metabolic genes. Among the 307 most frequently used CNS drugs, anxiolytics represent 11.40%, hypnotics and sedatives 21.17%, antidepressants 20.53%, and anti-dementia drugs 1–2%. About 90% of CNS drugs use CYP enzymes as major metabolic pathways. CNS drugs are major substrates of CYP3A4 (71%), CYP3A5 (37%), CYP2D6 (60%), CYP2C19 (45%), and CYP1A2 enzymes (44%); inhibitors of CYP3A4 (22%), CYP2D6 (23%), CYP2C19 (20%), CYP1A2 (17%), and CYP2C9 (15%); and inducers of CYP2C9 (9%), CYP2D6 (7%), CYP3A4 (5%), CYP1A2 (4.5%), CYP2A6 (4.5%), and CYP2B6 (3.7%). Major transporters of CNS drugs are ABCB1 (29%), SLCA1 (20%), SLC6A4 (20%), CLCNs (15%), SLC6A3 (12%), and SLC6A2 (11%) [26].

CYP2D6 variants are associated with 217 diseases, and 995 drugs are CYP2D6-related (218 major substrates, 174 minor substrates, 75 strong inhibitors, 183 moderate inhibitors, 32 weak inhibitors, and 18 inducers) [25]. Significant sex-related differences in the CYP2D6*4A/*41, *5/*5, *5/*6A, *41/*41 and *1A/*1xN genotypes have been identified in the Caucasian population. The number of CYP2D6-UMs is higher in males (6.16%) than in females (4.48%). These differences may contribute, in approximately 5–10% of the cases, to sex-related variability in the pharmacogenetic outcome and in the occurrence of DDIs and ADRs, as well [6,25,53,54].

The integration of CYP2D6-CYP2C9-CYP2C19 variants in trigenic clusters yields 134 genotypes and 33 phenotypes. The top five most frequent trigenic genotypes in the Iberian population are 1/1-1/1-1/1 (22.91%), 1/1-1/2-1/1 (10.45%), 1/1-1/1-1/2 (9.42), 1/4-1/1-1/1 (8.82%), and 1/4-1/2-1/1 (4.81%). Over 115 genotypes exhibit a frequency below 1%, with significant differences between females and males. Approximately 55% of trigenic phenotypes show a frequency below 1%, and only 23.55% of the subjects are extensive metabolizers for the three (2D6-2C9-2C19) enzymes [4,23].

About 25% of our patients with moderate-to-severe depression were treated with fluoxetine, paroxetine, or sertraline. Over 80% of these patients showed a net improvement in their depressive condition. However, these positive results cannot only be attributed to the effect of antidepressants, but also to the complementary treatments for their concomitant ailments. Meta-analyses of double-blind randomized controlled trials comparing antidepressants vs. placebo for depression in AD revealed inefficacy in most cases with different drugs (sertraline, mirtazapine, imipramine, fluoxetine, and clomipramine) [55].

The mechanisms underlying depression in dementia remain unclarified [56]. Genetic and environmental factors are potentially involved [44,57,58,59,60,61,62]. For instance, an increase in depression and anxiety symptoms have been reported in nursing homes during the coronavirus pandemic [63]. About 60% of depressive patients are receiving an inappropriate medication according to their pharmacogenetic background [64,65], and community psychiatrists and pharmacists are more accurate in their psychotropic prescriptions when they know the CYP profile of their patients [65,66,67].

### 3.4. Pharmacogenetics of Antidepressants

Antidepressants are associated with the pharmacogenetic activity of over 600 genes. The different pharmacological categories of antidepressants (non-selective monoamine reuptake inhibitors, selective serotonin reuptake inhibitors, non-selective monoamine oxidase (MAO) inhibitors, and other chemical modalities) are substrates, inhibitors, or inducers of 40, 22, and 9 enzyme/protein gene products, respectively, and are transported by 13 different protein transporters. Antidepressants are major substrates of CYP2D6 (86%), CYP3A4 (72%), CYP2C19 (60%), CYP1A2 (57%), CYP2C9 (34%), UGT1A4 (29%), and UGT1A3 (25%); inhibitors of CYP2D6 (69%), CYP3A4 (55%), CYP1A2 (45%), CYP2C19 (45%), CYP2C9 (34%), SLC6A4 (32%), MAOA (29%), MAOB (29%), and ABCB1 (25%); and inducers of CYP3A4 (5%), CYP1A2 (5%), CYP2B6 (5%), CYP2C9 (3%), CYP2C19 (3%), CYP2D6 (3%), and ABCB1 (3%). Major transporters of antidepressants are SLC6A4 (62%), ABCB1 (55%), and SLC6A2 (40%). For instance, Sertraline is associated with 31 pharmagenes and Fluoxetine with 28 genes potentially involved in the pharmacogenetic outcome [25,26].

Fluoxetine is a major substrate of CYP2D6 and CYP2C9; a minor substrate of CYP1A2, CYP2B6, CYP2E1, and CYP3A4; a strong inhibitor of CYP2D6, a moderate inhibitor of CYP1A2 and CYP2C19, and a weak inhibitor of CYP2B6, CYP2C9, and CYP3A4 [25,54]. Paroxetine is a major substrate of CYP2D6; a strong inhibitor of CYP2D6; a moderate inhibitor of CYP2B6 and a weak inhibitor of CYP1A2, CYP2C8, CYP2C9, CYP2C19, and CYP3A4 [25,54]. Sertraline is a major substrate of CYP2D6 and CYP2C19; a minor substrate of CYP2A6, CYP2B6, CYP2C9, and CYP3A4; a moderate inhibitor of CYP2C19, CYP2D6, and CYP3A4; and a weak inhibitor of CYP1A2, CYP2C8, and CYP2C9 [25,54]. CYP2C19 and CYP2D6 variants affect the occurrence of ADRs in patients treated with selective serotonin reuptake inhibitors (SSRIs)(citalopram, escitalopram, sertraline, fluvoxamine, fluoxetine, paroxetine), including anxiety associated with CYP2D6, and ECG prolonged QT intervals associated with CYP2C19 [68]. The serotonin receptor 1A (HTR1A) rs878567 and CYP2C19 rs12248560 gene variants are associated with depression severity [69]. Angiotensin-converting enzyme (ACE) variants influence mood in AD [49]. The co-administration of ACE inhibitors and statins with antidepressants may affect therapeutic outcomes [70]. In our case, we did not observe any ADRs in the patients treated with small doses of enalapril, atorvastatin, and SSRIs.

### 3.5. Pharmacogenetics of Anxiolytics

The neurochemical mechanisms of anxiety in dementia are unknown. Subjective cognitive decline and neuropsychiatric symptoms are associated with brain structural alterations and APOE-4 [71,72]. Benzodiazepines are commonly prescribed for ameliorating anxiety symptomatology; however, benzodiazepines contribute to cognitive and psychomotor dysfunction [73,74]. CYP enzymes participate in the metabolism of over 92% of benzodiazepines. About 70% of these drugs are major substrates of CYP3A4, followed by CYP2C19 (41%), CYP3A5 (38%), CYP2D6 (36%), CYP2C9 (30%), CYP1A2 (27%), CYP2B6 (19%), UGT1A4 (14%), UGT2B15 (11%), and UGT1A1, UGT1A3, UGT1A6, UGT1A10, and UGT2B7 (8%); only 10% are inhibitors of CYP3A4 and CYP2C9; 8% are inducers of CYP3A4, and about 5% are inducers of CYP1A2, CYP2A6, CYP7A1, and ABCC2. Over 50% of these drugs are transported by proteins of the CLCN family, 16% are transported by ABCB1, 9% by NQ1I2, and 5% by ABCC2, KCNE1, KCNH2, and SLCO1B1 [2,26]. Alprazolam and Lormetazepam are major substrates of CYP3A4/4 and minor substrates of CYP1A1, CYP1A2, CYP2B6, CYP2C9, CYP2C19, and CYP2D6 [25]. In our study, they show benefit with small doses by alleviating anxiety without significant disturbance in cognitive and psychomotor functions. There is unnecessary over-prescription of psychotropic drugs in patients with dementia, which contribute to accelerated cognitive decline and increased accidentality and mortality. Pharmacogenomics of AD has proven to be useful for the prediction of therapeutic outcome, discrimination of responders vs. non-responders, and prevention of ADRs and unwanted DDIs [2,23,26,27].

Our study demonstrates that a multifactorial regime, covering biochemical and metabolic deficiencies, together with neuroprotectants and adjusted psychotropic medication, can be beneficial for emotional stability in AD patients, and that APOE-4 carriers and CYP-related PMs and UMs, representing over 40% of the AD population, deserve special attention for their vulnerability and poor response to current treatments.

## 4. Materials and Methods 

### 4.1. Patients and Clinical Protocol

Patients for this retrospective study were recruited from the CIBE database at the International Center of Neuroscience and Genomic Medicine (period: 2000–2010; follow-up: 2000–2020). This cohort includes patients (*N* = 1006; age: 67.71 ± 9.62 years; range: 50–96 years) of both sexes (591 Females; age: 67.52 ± 9.68 years; range: 50–96 years; 415 Males; age: 67.50 ± 9.54) with the diagnosis of neurocognitive disorder (NCD)-AD (331.0 (G30.9) (DSM-V/NINCDS-ADRDA criteria). All patients underwent the following protocol: (i) Clinical examination, (ii) blood and urine analyses, (iii) neuropsychological assessment (MMSE [75], ADAS [76], HARS [77], HDRS [78]), (iv) cardiovascular evaluation (EKG), (v) structural neuroimaging (brain MRI), (vi) functional neuroimaging (brain mapping, brain optical topography), (vii) genetic assessment, and (viii) pharmacogenetic profiling, as reported in previous pharmacogenetic studies [50,51].

The patients received, for one year, a multifactorial therapy integrated by CDP-choline (500 mg/day, p.o.) (choline donor and intermediate metabolite in DNA synthesis and repair), Piracetam (1600 mg/day, p.o.) (nootropic drug), Sardilipin (E-SAR-94010) (250 mg, t.i.d.)(nutraceutical with lipid-lowering effects and anti-atherosclerotic properties), and Animon Complex^®^ (2 capsules/day)(a nutraceutical compound integrated by a purified extract of Chenopodium quinoa (250 mg), ferrous sulphate (38.1 mg equivalent to 14 mg of iron), folic acid (200 µg), and vitamin B12 (1 µg per capsule). About 5% of patients received Donepezil (5 mg/day). Patients with chronic deficiency of iron (<35 µg/mL)(4.55%), folic acid (<3 ng/mL)(5.60%), or vitamin B12 (<170 pg/mL) (4.85%) received an additional supplementation of iron (80 mg/day), folic acid (5 mg/day), and B complex vitamins (B1, 15 mg/day; B2, 15 mg/day; B6, 10 mg/day; B12, 10 µg/day; nicotinamide, 50 mg/day), respectively, to maintain stable levels of serum iron (50–150 µg/mL), folic acid (5–20 ng/mL), and vitamin B12 levels (500–1000 pg/mL) in order to avoid the negative influence of these metabolic factors on cognition and mood. Patients with hypertension (>150/85 mmHg) (35%) received enalapril (5–20 mg/day, p.o.); patients with hypercholesterolemia (>220 mg/dL) (46%) received atorvastatin (10–20 mg/day); patients with diabetes (glucose >105 mg/dL) (28%) received metformin (850–1700 mg/day, p.o.); and patients (<3%) with other ailments (e.g., hypothyroidism and hyperuricemia) received the appropriate treatment according to their medical condition. Patients with moderate-to-severe depression (25%) received Fluoxetine (20 mg/day), Paroxetine (20 mg/day), or Sertraline (50 mg/day); and patients with agitation and/or moderate-to-severe anxiety (18%) received Alprazolam (0.5–1.0 mg/day) or Lorazepam (1–2 mg/day) for one month. Less than 3% of patients required neuroleptics (Risperidone, Quetiapine, Levomepromazine, Haloperidol) for the treatment of severe BDs. Blood pressure, psychometric assessment (Mini-Mental State Examination, MMSE; Alzheimer’s Disease Assessment Scale, ADAS; Hamilton Rating Scale-Depression, HAM-D; Hamilton Rating Scale-Anxiety, HAM-A), and blood parameters (Table 1) were evaluated prior to treatment (baseline) and after 1, 3, 6, 9, and 12 months of treatment. In this study, we analyzed parameters at baseline and after one month of treatment. Results on the pharmacogenetics of cognition for one year can be found elsewhere [4,49,50,51]. All patients and/or their legal representatives provided informed consent for genotyping, clinical assessment, and treatment before they participated in the study. The study was conducted in accordance with the Declaration of Helsinki, and the protocol was approved by the Ethics Committee of the EuroEspes Biomedical Research Center (Project identification code: CDN-AD-DEP-01-99-10).

### 4.2. Genotyping

DNA was extracted from peripheral blood using Qiagen extraction columns (Qiagen, Hilden, Germany). SNPs in 18 genes encoding for proteins associated with AD pathogenesis and a panel of 113 SNPs and 3 copy number variations (CNVs) in 60 genes encoding for pharmacogenetics-related genes (metabolic, transporter, mechanistic, pleiotropic genes) were genotyped (Table 4 and Table 5). Genes of interest for this study were the following: (i) APOE (rs7412, c.4070C > T, Cys158Arg (*2); rs429358, c.3932T > C, Cys112Arg (*4); (ii) CYP2C19 (Cytochrome P450 family 2 subfamily C member 19) (rs4244285, c.681G > A (*2A); rs12248560, g.-806C > T (*17)); (iii) CYP2C9 (Cytochrome P450 family 2 subfamily C member 9)(rs1799853, c.430C > T (*2A); rs1057910, c.1075A > C (*3A); rs28371686, c.1080C > A (*5A); rs9332131, c.817delA (*6A); rs7900194, c.449G > T (*8A); rs28371685, c.1003C > T (*11A); and (iv) CYP2D6 (Cytochrome P450 family 2 subfamily D member 6)(rs35742686, g.2549delA (*3A); rs3892097, g.1846G > A (*4A); rs5030655, g.1707T>del (*6A); CNV, CYP2D6 indel (*5, *1 × N); rs28371725, g.2988G > A (*41). Real-Time Polymerase Chain Reaction (RT-PCR) amplification was performed using TaqMan assays for SNPs using StepOne Plus Real -Time PCR System (Life Technologies) and/or TaqMan^®^ OpenArray^®^ DNA microchips for QuantStudioTM 12K Flex Real-Time PCR System. OpenArray^®^ genotyping analysis was performed using the Genotyper software (Thermo Fisher Scientific, Waltham, MA, USA).

### 4.3. Statistical Analysis

Data were analyzed by using IBM SPSS Statistics 20 (IBM Corp., Armonk, NY, USA) and SigmaPlot 10.0 (SYSTAT Software Inc., San Jose, CA, USA). Comparisons between groups were studied by t-Test, Mann–Whitney Rank Sum Test, Chi Square without Yates correction and Fisher exact, and Pearson Correlation Analysis (Nonlinear Regression, Durbin–Watson Statistic, Normality Test, Constant Variance Test, 95% Confidence), when appropriate. In studies of correlation analysis, all cases have been ordinated from the lowest to the highest values for maximum differentiation in the figures. All values are expressed as mean ± SD, and the degree of significance is considered when *p* < 0.05.

## 5. Conclusions

Major conclusions from this study include the following: (i) Biochemical, hematological, and metabolic differences may contribute to changes in drug efficacy and safety (tentative contribution: 5–20% that depends on the number of drugs involved); (ii) anxiety and depression are more frequent and severe in females than males with dementia; (iii) cardiovascular disorders and associated risk factors are more frequent in males than females; however, vascular problems appear to be unrelated to mood disorders in dementia; (iv) both females and males respond similarly to a multifactorial regime, showing significant improvements in anxiety and depression; (v) APOE-3 carriers are the best responders and APOE-4 carriers tend to be the worst responders to conventional treatments in combination regimes; (vi) among CYP2D6, CYP2C19, and CYP2C9 geno-phenotypes, NMs and IMs are significantly better responders than PMs and UMs to therapeutic interventions that modify anxiety and depression phenotypes; and (vii) APOE-4 carriers and CYP-related PMs and UMs deserve special attention for their vulnerability and poor response to current treatments.

The main conclusions related to pharmacogenetics should be interpreted with caution taking into account the polypharmacy received by dementia patients and assuming that the pharmacogenetic response depends on many more genes than those discussed in this study.

## Figures and Tables

**Table 1:** Sample features.

Parameter	Total	Females	Males	Differences
*N*	1006	591	415	
Age (Years)	67.51 ± 9.62	67.52 ± 9.68	67.50 ± 9.54	*p* = 0.97
Systolic Blood Pressure (mm Hg)	138.70 ± 20.06	137.40 ± 19.83	140.54 ± 1.96	*p* < 0.02
Diastolic Blood Pressure (mm Hg)	79.74 ± 10.81	79.25 ± 10.70	80.45 ± 10.94	*p* < 0.04
Pulse (bpm)	66.67 ± 11.71	68.10 ± 11.28	64.86 ± 12.06	*p* < 0.001
Weight (Kg)	72.01 ± 13.66	66.84 ± 12.20	79.49 ± 12.12	*p* < 0.001
Hight (m)	1.60 ± 0.09	1.54 ± 0.06	1.67 ± 0.06	*p* < 0.001
BMI (Kg/m^2^)	28.14 ± 4.57	28.01 ± 5.11	28.31 ± 3.63	*p* < 0.03
Glucose (mg/dL)	100.01 ± 24.91	96.47 ± 22.56	105.03 ± 27.15	*p* < 0.001
Cholesterol (mg/dL)	225.69 ± 46.03	235.02 ± 44.92	212.43 ± 44.35	*p* < 0.001
HDL-Cholesterol (mg/dL)	54.36 ± 14.81	59.42 ± 14.93	47.18 ± 11.27	*p* < 0.001
LDL-Cholesterol (mg/dL)	148.28 ± 39.75	153.55 ± 40.23	140.80 ± 37.88	*p* < 0.001
Triglycerides (mg/dL)	115.42 ± 69.97	108.14 ± 55.18	125.77 ± 85.79	*p* < 0.005
Urea (mg/dL)	42.49 ± 12.44	41.30 ± 11.76	44.20 ± 13.16	*p* < 0.001
Creatinine (mg/dL)	0.87 ± 0.21	0.79 ± 0.17	0.98 ± 0.22	*p* < 0.001
Uric Acid (mg/dL)	4.38 ± 1.97	3.77 ± 1.20	5.26 ± 2.47	*p* < 0.001
Total Protein (g/dL)	6.89 ± 0.46	6.90 ± 0.41	6.88 ± 0.52	*p* = 0.99
Albumin (g/dL)	4.32 ± 0.32	4.31 ± 0.27	4.35 ± 0.38	*p* < 0.05
Calcium (mg/dL)	9.20 ± 0.49	9.24 ± 0.46	9.14 ± 0.53	*p* < 0.02
Phosphorus (mg/dL)	3.42 ± 0.54	3.52 ± 0.52	3.27 ± 0.52	*p* < 0.001
GOT/ASAT (IU/L)	22.44 ± 19.95	22.57 ± 23.45	22.26 ± 13.53	*p* = 0.66
GPT/ALAT (IU/L)	23.97 ± 20.75	22.57 ± 21.31	25.95 ± 19.79	*p* < 0.001
GGT (IU/L)	30.55 ± 39.93	26.85 ± 37.52	35.79 ± 42.61	*p* < 0.001
Alkaline Phosphatase IU/L)	74.69 ± 28.30	77.12 ± 29.20	71.23 ± 26.63	*p* < 0.001
Bilirubin (mg/dL)	0.79 ± 2.04	0.78 ± 2.64	0.81 ± 0.41	*p* < 0.001
CPK (IU/L)	92.48 ± 124.94	88.61 ± 153.17	97.98 ± 66.60	*p* < 0.001
LDH (IU/L)	292.63 ± 62.05	303.52 ± 63.80	277.12 ± 56.01	*p* < 0.001
Na^+^ (mEq/L)	142.40 ± 2.32	142.50 ± 2.15	142.26 ± 2.54	*p* = 0.17
K^+^ (mEq/L)	4.37 ± 0.37	4.30 ± 0.36	4.46 ± 0.36	*p* < 0.001
Cl^−^ (mEq/L)	104.46 ± 2.47	104.64 ± 2.37	104.20 ± 2.60	*p* < 0.01
Fe^2+^ (µg/dL)	87.42 ± 33.67	82.89 ± 31.78	93.81 ± 35.23	*p* < 0.001
Ferritin (ng/mL)	114.81 ± 125.83	83.87 ± 95.10	158.58 ± 149.01	*p* < 0.001
Folate (ng/mL)	7.24 ± 3.86	7.50 ± 3.92	6.87 ± 3.76	*p* < 0.003
Vitamin B_12_ (pg/mL)	502.17 ± 297.90	516.45 ± 302.12	481.80 ± 290.92	*p* < 0.003
TSH (µIU/mL)	1.48 ±1.77	1.56 ± 2.03	1.37 ± 1.29	*p* < 0.01
T4 (ng/mL)	0.88 ± 0.18	0.88 ± 0.17	0.89 ± 0.18	*p* = 0.47
RBC (x10^6^/µL)	4.63 ± 0.44	4.48 ± 0.37	4.84 ± 0.45	*p* < 0.001
HCT (%)	42.06 ± 4.05	40.59 ± 3.57	44.16 ± 3.75	*p* < 0.001
Hb (g/dL)	14.04 ± 1.32	13.51 ± 1.06	14.80 ± 1.29	*p* < 0.001
VCM (fL)	90.84 ± 5.02	90.45 ± 5.05	91.38 ± 4.92	*p* < 0.001
HCM (pg)	30.38 ± 1.89	30.20 ± 1.90	30.64 ± 1.84	*p* < 0.001
CHCM (g/dL)	33.41 ± 1.21	33.34 ± 1.45	33.51 ± 0.74	*p* < 0.02
ADE (RDW)(%)	13.04 ± 1.41	13.06 ± 1.60	13.00 ± 1.09	*p* = 0.74
WBC (x10^3^/µL)	6.22 ± 1.86	6.02 ± 1.90	6.51 ± 1.76	*p* < 0.001
%Neutrophils	59.64 ± 9.38	59.62 ± 9.23	59.66 ± 9.61	*p* = 0.98
%Lymphocytes	30.01 ± 8.54	30.54 ± 8.47	29.25 ± 8.59	*p* < 0.01
%Monocytes	7.33 ± 2.04	7.14 ± 2.00	7.61 ± 2.06	*p* < 0.001
%Eosinophils	2.75 ± 2.06	2.61 ± 2.93	2.95 ± 2.03	*p* < 0.001
%Basophils	0.53 ± 0.28	0.49 ± 0.19	0.59 ± 0.37	*p* < 0.001
PLT (x10^3^/µL)	225.13 ± 62.29	237.40 ± 59.71	207.61 ± 61.78	*p* < 0.001
VPM (fL)	8.36 ± 0.91	8.27 ± 0.89	8.48 ± 0.96	*p* < 0.001
ESR (mm/hr)	19.08 ± 15.75	21.66 ± 15.38	15.37 ± 15.55	*p* < 0.001
EKG	*N*: 51.18%; AN: 47.82%	*N*: 56.64% AN: 43.36%	*N*: 45.00% AN: 55.00%	*p* < 0.001 *
MMSE Score (30)	23.16 ± 5.95	22.42 ± 5.88	24.22 ± 5.90	*p* < 0.001
ADAS-Cog	19.14 ± 12.88	19.64 ± 12.71	18.40	*p* = 0.06
ADAS-Non Cog	4.60 ± 3.82	5.17 ± 4.02	3.77 ± 3.36	*p* < 0.001
ADAS-Total	22.78 ± 15.22	23.76 ± 15.07	21.36 ± 15.35	*p* < 0.002
HARS	11.44 ± 5.41	12.49 ± 5.63	9.94 ± 4.69	*p* < 0.001
HDRS	10.11 ± 5.21	10.85 ± 5.33	9.05 ± 4.84	*p* < 0.001

Data: mean ± SD; * Pearson’s Chi-squared test with Yates continuity correction.

**Table 2:** Geno-phenotype-related anxiety changes in patients with Alzheimer’s Disease treated with a multifactorial therapeutic regime.

GenoPhenotype	*N*	%	HARS-0	HARS-1	*p* Value
**APOE-2/2**	2	0.20	15.5 ± 10.6	11.50 ± 12.02	*p* = 0.70
**APOE-2/3**	83	8.54	11.13 ± 4.96 ^(1)^	10.03 ± 3.98 ^(7–8)^	*p* = 0.19
**APOE-2/4**	14	1.44	15.00 ± 4.64 ^(2–4)^	10.35 ± 3.50	*p* < 0.006
**APOE-3/3**	601	61.83	11.72 ± 5.27 ^(5–6)^	9.83 ± 4.34	*p* < 0.001
**APOE-3/4**	238	24.49	10.72 ± 5.41	9.33 ± 4.22	*p* < 0.001
**APOE-4/4**	34	3.50	9.85 ± 5.47	8.50 ± 3.77	0.39
**CYP2D6-NM**	343	57.74	11.30 ± 5.31 ^(9)^	9.51 ± 4.07	*p* < 0.001
**CYP2D6-IM**	182	30.64	11.02 ± 5.06 ^(10)^	9.07 ± 3.57	*p* < 0.001
**CYP2D6-PM**	33	5.56	9.75 ± 7.01	8.48 ± 3.89	0.76
**CYP2D6-UM**	36	6.06	10.05 ± 4.52	8.94 ± 3.02	0.37
**CYP2C19-NM**	423	71.21	11.41 ± 5.67	9.42 ± 3.92	*p* < 0.001
**CYP2C19-IM**	153	25.76	10.59 ± 5.12	9.03 ± 3.84	*p* < 0.01
**CYP2C19-PM**	8	1.35	9.65 ± 4.80	8.00 ± 3.11	0.43
**CYP2C19-UM**	10	1.68	13.50 ± 6.06	11.30 ± 4.11	0.35
**CYP2C9-NM**	363	62.26	11.21 ± 5.35	9.23 ± 3.93	*p* < 0.001
**CYP2C9-IM**	190	32.59	10.84 ± 5.26	9.26 ± 3.72	*p* < 0.01
**CYP2C9-PM**	30	5.15	10.66 ± 5.20	9.50 ± 3.95	0.33

APOE: (1) *p* < 0.007 2/3-HARS0 vs. 2/4-HARS0; (2) *p* < 0.01 2/4-HARS0 vs. 3/3-HARS0; (3) *p* < 0.002 2/4-HARS0 vs. 3/4-HARS0; (4) *p* < 0.001 2/4-HARS0 vs. 4/4-HARS0; (5) *p* < 0.005 3/3-HARS0 vs. 3/4-HARS0; (6) *p* < 0.02 3/3-HARS0 vs. 4/4-HARS0; (7) *p* < 0.05 2/3-HARS1 vs. 3/4-HARS1; (8) *p* < 0.05 2/3-HARS1 vs. 4/4-HARS1. CYP2D6: (9) *p* < 0.03 NM-HARS0 vs. PM-HARS0; (10) *p* < 0.05 IM-HARS0 vs. PM-HARS0.

**Table 3:** Geno-phenotype-related depression changes in patients with Alzheimer’s Disease treated with a multifactorial therapeutic regime.

GenoPhenotype	*N*	%	HDRS-0	HDRS-1	*p* Value
**APOE-2/2**	2	0.20	10.00 ± 5.65	7.50 ± 7.77	*p* = 0.13
**APOE-2/3**	83	8.54	10.50 ± 4.91 ^(1–2)^	9.39 ± 4.55 ^(5–6)^	*p* = 0.13
**APOE-2/4**	14	1.44	11.14 ± 4.44 ^(3)^	8.64 ± 4.19	*p* = 0.18
**APOE-3/3**	601	61.83	10.19 ± 5.17 ^(4)^	8.61 ± 4.21 ^(7)^	*p* < 0.001
**APOE-3/4**	238	24.49	9.76 ± 5.24	8.33 ± 4.16	*p* < 0.003
**APOE-4/4**	34	3.50	8.58 ± 4.34	7.08 ± 3.76	0.13
**CYP2D6-NM**	343	57.74	9.98 ± 4.91 ^(8)^	8.55 ± 4.18	*p* < 0.001
**CYP2D6-IM**	182	30.64	9.54 ± 4.86 ^(9)^	8.16 ± 3.68	*p* < 0.006
**CYP2D6-PM**	33	5.56	9.24 ± 6.55	7.36 ± 4.13	0.33
**CYP2D6-UM**	36	6.06	9.44 ± 4.79	7.97 ± 3.43	0.25
**CYP2C19-NM**	423	71.21	10.06 ± 5.12 ^(10–11)^	8.44 ± 4.00	*p* < 0.001
**CYP2C19-IM**	153	25.76	9.16 ± 4.78	7.92 ± 4.01	*p* < 0.02
**CYP2C19-PM**	8	1.35	8.75 ± 3.88	8.00 ± 2.33	0.64
**CYP2C19-UM**	10	1.68	10.80 ± 5.18	9.20 ± 3.12	0.41
**CYP2C9-NM**	363	62.26	9.97 ± 5.18	8.47 ± 4.81	*p* < 0.001
**CYP2C9-IM**	190	32.59	9.46 ± 4.80	8.10 ± 3.82	*p* < 0.009
**CYP2C9-PM**	30	5.15	9.00 ± 4.02	7.46 ± 3.35	0.14

APOE: (1) *p* < 0.05 2/3-HDRS0 vs. 3/4-HDRS0; (2) *p* < 0.05 2/3-HDRS0 vs. 4/4-HDRS0; (3) *p* < 0.05 2/4-HDRS0 vs. 4/4-HDRS0; (4) *p* < 0.05 /3-HDRS0 vs. 4/4-HDRS0; (5) *p* < 0.03 2/3-HDRS1 vs. 3/4-HDRS1; (6) *p* < 0.01 2/3-HDRS1 vs. 4/4-HDRS1; (7) *p* < 0.05 3/3-HDRS1 vs. 4/4-HDRS1. CYP2D6: (8) *p* < 0.03 NM-HDRS0 vs. PM-HDRS0; (9) *p* < 0.05 IM-HDRS0 vs. PM-HDRS0. CYP2C19: (10) *p* < 0.05 NM-HDRS0 vs. IM-HDRS0; (11) *p* < 0.05 NM-HDRS0 vs. PM-HDRS0.

**Table 4:** Selected Alzheimer’s disease pathogenic genes.

Gene Symbol	Gene Name	Locus	dbSNP	Polymorphism	Assay ID
*A2M*	alpha-2-macroglobulin	12p13.31	rs669	c. 2998A > G, V1000I	C____517658_10
*ABCA7*	ATP binding cassette subfamily A member	19p13.3	rs3764650	c. 1622+115T > G	C__27478162_10
*ACE*	angiotensin I converting enzyme	17q23.3	rs4332	c. 496-66T > C	C__11942538_20
*APOE*	apolipoprotein E	19q13.32	rs429358	c. 3932T > C, Cys112Arg	C___3084793_20
			rs7412	c. 4070C > T, Cys158Arg	C____904973_10
*BIN1*	bridging integrator 1	2q14.3	rs744373	g. 127137039A > G	C___1042213_10
*C9ORF72*	chromosome 9 open reading frame 72	9p21.2	rs3849942	g. 27543283T > C	C__27515934_20
*CLU*	clusterin	8p21.1	rs11136000	c. 247-478A > G	C__11227737_10
*CPZ*	carboxypeptidase Z	4p16.1	rs7436874	g. 8649098C > T	C____506568_20
*CR1*	complement C3b/C4b receptor 1	1q32.2	rs3818361	c. 4946-54A > G	C__25598588_10
*DISC1* *LHFPL6* *MS4A4E*	disrupted in schizophrenia 1 LHFPL tetraspan subfamily member 6 membrane spanning 4-domains A4E	1q42.2 13q13.3-q14.11 11q12.2	rs16856202	c. 2242-7030T > G	C__33950435_10
			rs7995844	g. 39298100G > A	C__29428261_10
			rs670139	c. 279-2443C > A	C___7512835_20
*MS4A6A*	membrane spanning 4-domains A6A	11q12.2	rs610932	c. *149 + 175A > C	C__27161626_10
*NOS3*	nitric oxyde synthse 3	7q36.1	rs1799983	c. 894G > T, E298D	C___3219460_20
*PICALM*	phosphatidylinositol binding clathrin assembly protein	11q14.2	rs3851179	g. 85868640T > C	C___8748810_10
*PRNP*	prion protein	20p13	rs1799990	c. 385A > G, M129V	C___2969398_10
*PSEN1*	presenilin 1	14q24.2	rs165932	c. 856+16G > T	C____579315_20
*TNF*	tumor necrosis factor	6p21.33	rs1800629	c. -308G > A	C___7514879_10

**Table 5:** Phase I Metabolic Genes.

Gene Symbol	Gene Name	Locus	dbSNP	Polymorphism	Assay ID
*CYP1A1*	cytochrome P450 family 1 subfamily A member 1	15q24.1	rs1378942	c. −66 + 2306C > A	C___1642446_10
*CYP1A2*	cytochrome P450 family 1 subfamily A member 2	15q24.1	rs2069514	g. −3860G > A	C__15859191_30
			rs35694136	g. −2467delT	C__60142977_10
			rs762551	g. −163C > A	C___8881221_40
*CYP1B1*	cytochrome P450 family 1 subfamily B member 1	2p22.2	rs1056836	c. 1294C > G; p. Leu432Val	C___3099976_30
*CYP2A6*	cytochrome P450 family 2 subfamily A member 6	19q13.2	rs28399433	g.−48T > G	C__30634332_10
*CYP2B6*	cytochrome P450 family 2 subfamily B member 6	19q13.2	rs3745274	c.516G > T; p.Gln172His	C___7817765_60
*CYP2C19*	cytochrome P450 family 2 subfamily C member 19	10q23.33	rs12248560	g. −806C > T	C____469857_10
			rs4244285	c.681G > A	C__25986767_70
*CYP2C9*	cytochrome P450 family 2 subfamily C member 9	10q23.33	rs1057910	c. 1075A > C	C__27104892_10
			rs1799853	c. 430C > T	C__25625805_10
			rs28371685	c. 1003C > T	C__30634132_70
			rs28371686	c. 1080C > A	C__27859817_40
			rs7900194	c. 449G > T	C__25625804_10
			rs9332131	c. 817delA	C__32287221_20
*CYP2D6*	cytochrome P450 family 2 subfamily D member 6	22q13.2	indel	Gene duplication/deletion	Hs00010001_cn
			rs28371725	g. 2988G > A	C__34816116_20
			rs35742686	g. 2549delA	C__32407232_50
			rs3892097	g. 1846G > A	C__27102431_D0
			rs5030655	g. 1707T > del	C__32407243_20
*CYP2E1*	cytochrome P450 family 2 subfamily E member 1	10q26.3	rs3813867	g. −1293G > C	C___2431875_10
			rs6413420	g. −71G > T	C__25594209_10
*CYP3A4*	cytochrome P450 family 3 subfamily A member 4	7q22.1	rs2242480	g. 20230G > A	C__26201900_30
			rs35599367	g. 20493C > T	C__59013445_10
*CYP3A5*	cytochrome P450 family 3 subfamily A member 5	7q22.1	rs776746	g. 6986A > G	C__26201809_30
*CYP4F2*	cytochrome P450 family 4 subfamily F member 2	19p13.12	rs2108622	c. 1297G > A	C__16179493_40
*DPYD*	dihydropyrimidine dehydrogenase	1p21.3	rs3918290	c. 1905+1G > A/C	C__30633851_20
			rs55886062	c. 1679T > G; p.Ile560Ser	C__11985548_10
			rs67376798	c. 2846A > T; p. Asp949Val	C__27530948_10
*G6PD*	glucose-6-phosphate dehydrogenase	Xq28	rs1050828	c. 202G > A; p.Val68Met	C___2228686_20
			rs5030868	c. 563C > T; Ser188Phe	C___2228708_20
*MAOB*	monoamine oxidase B	Xp11.3	rs1799836	c. 1300−36A > G	C___8878790_10

## Data Availability

Data supporting reported results can be found in the CIBE Database at EuroEspes International Center of Neuroscience and Genomic Medicine. www.euroespes.com.
